# Can a bleaching toothpaste containing Blue Covarine demonstrate the same bleaching as conventional techniques? An *in vitro*, randomized and blinded study

**DOI:** 10.1590/1678-775720150268

**Published:** 2015

**Authors:** Andréa Abi Rached DANTAS, Janaina Freitas BORTOLATTO, Ávery RONCOLATO, Hugo MERCHAN, Michael Christopher FLOROS, Milton Carlos KUGA, Osmir Batista de OLIVEIRA

**Affiliations:** 1 - Universidade Estadual Paulista, Faculdade de Odontologia de Araraquara, Departamento de Odontologia Restauradora, Araraquara, SP, Brazil.; 2 - Trent University, Department of Physics & Astronomy and Department of Chemistry, Center for Biomaterials Research, Peterborough, Canada.

**Keywords:** Tooth bleaching, Dentifrices, Color, Efficacy, Spectrophotometers

## Abstract

**Objective:**

The purpose of this *in vitro* study was to compare the efficacy of a bleaching toothpaste containing Blue Covarine vs. conventional tooth bleaching techniques using peroxides (both in-office and at-home).

**Material and Methods:**

Samples were randomly distributed into five experimental groups (n=15): C - Control; BC – Bleaching toothpaste containing Blue Covarine; WBC – Bleaching toothpaste without Blue Covarine; HP35 - In-office bleaching using 35% hydrogen peroxide; and CP10 – At-home bleaching with 10% carbamide peroxide. The dental bleaching efficacy was determined by the color difference (ΔE), luminosity (ΔL), green-red axis (Δa), and blue-yellow axis (Δb). The CIELab coordinates were recorded with reflectance spectroscopy at different times: T0 - baseline, T1 – immediately after bleaching, T2 - 7 days, T3 - 14 days, and T4 - 21 days after the end of treatments. Data were analyzed by a repeated measures mixed ANOVA and post hoc Bonferroni test, with a significance level of 5%.

**Results:**

No significant differences were found between the treatment groups C, BC, and WBC. The groups HP35 and CP10 showed significantly higher whitening efficacy than groups C, BC, and WBC.

**Conclusions:**

There were no significant differences in the whitening efficacy between a Blue Covarine containing toothpaste, a standard whitening toothpaste, and a control. Neither of the whitening toothpastes tested were as effective as in-office or at-home bleaching treatments.

## INTRODUCTION

Conventional tooth bleaching techniques are based on the action of free radicals from the reaction of hydrogen or carbamide peroxides with the tooth structure^10^. However, free radicals can also cause adverse biological reactions in addition to the desired aesthetic result^3^ ^-^ ^5^ ^,^ ^17^ ^,^ ^19^. During tooth bleaching, the most common negative effect is trans- and post-operative tooth sensitivity from peroxide based free radicals^4^ ^,^ ^8^ ^,^ ^16^. According to Dahl and Pallensen^4^ (2003), tooth sensitivity occurs both in at-home (15 to 65% incidence) and in-office tooth bleaching (67 to 78% incidence) and it represents a major concern with this technique.

To avoid these risks, bleaching toothpastes have been formulated to act removing and/or controlling the extrinsic staining by the action of abrasives, surfactants, polyphosphates, and enzymes^12^. A new toothpaste, which claims to have a superior bleaching effect, has recently been introduced to the market. This toothpaste acts to alter the perception of tooth whiteness by an optical effect, as opposed to the removal or alteration of pigments within the teeth that change the color of the tooth structure. Furthermore, this toothpaste contains modified silica particles, an abrasive that aids in removing extrinsic surface stains. This optical effect occurs by deposition of a thin blue film on the enamel surface^13^. The toothpaste contains Blue Covarine, a pigment which is uniformly deposited and retained on pellicle-coated tooth surfaces causing a color shift that ultimately induces an increase in the measurement and perception of tooth whiteness. By modifying the visual perception of tooth structure color through displacement of the axis b* from yellow to blue, an illusion of higher luminosity and bleaching is obtained^13^.

Despite the positive results reported in laboratory and clinical studies^2^ ^,^ ^12^ ^,^ ^13^, it has not been proven yet that this bleaching film can prevail over the color of the tooth structure in situations of higher aesthetic challenge, such as very yellow or gray teeth, or if under these conditions the final perception of tooth color will be the sum of the yellow and blue colors, or gray and blue, resulting in gray-blue or greenish teeth, respectively, and not in the desired bleaching effect. Furthermore, the efficacy of this bleaching toothpaste still has to be compared to conventional bleaching techniques.

This study was conducted to evaluate how a toothpaste containing Blue Covarine and modified silica is compared to traditional tooth bleaching techniques using peroxides. The null hypothesis we tested was if the average bleaching obtained with the toothpaste containing Blue Covarine and modified silica is similar to other bleaching treatments.

## MATERIAL AND METHODS

### Study design and sample preparation

A controlled laboratory, randomized, paired and blinded study was performed.

After prophylaxis, 90 bovine incisors were selected according to similar color, good structural integrity, and surface regularity. First, the roots of the teeth were removed using a low-speed, water-cooled diamond saw (Isomet 4000, Buehler Ltd., Lake Bluff, IL, USA). Then, the teeth were sectioned in mesiodistal and cervicoincisal directions to obtain dental blocks with dimensions of 6.0x6.0x2.0 mm. The samples were numbered and randomly assigned into experimental groups by random drawing, using Microsoft Excel 2010 (Microsoft, Redmond, WA, USA). Then, they were embedded in a neutral gray acrylic resin in sets of five dental blocks that were coded and stored in artificial saliva under stirring at a temperature of 36±1.0°C for a week prior to testing. The 15 experimental sets were randomly distributed into 5 experimental groups (n=15), according to the bleaching technique: in-office with 35% hydrogen peroxide - HP35 (Whiteness HP^®^ - FGM Produtos Odontológicos, Joinville, SC, Brazil), at-home using 10% carbamide peroxide - CP10 (Whiteness Perfect^®^ - FGM Produtos Odontológicos, Joinville, SC, Brazil); a Blue Covarine containing toothpaste - BC (Close Up White Now – Unilever Brazil, São Paulo, SP, Brazil), and a bleaching toothpaste without Blue Covarine - WBC (Colgate MaxWhite – Colgate Palmolive Brazil, São Paulo, SP, Brazil). The control group (C) did not receive any treatment. The artificial saliva was changed once a week for all samples.

### Sample size determination

The primary outcome of this study was the color difference determined by delta E (ΔE). The sample size calculation was based on data obtained from a previous pilot study using an in-office bleaching agent containing 35% hydrogen peroxide that lead to a ΔE value of ١١±2.0 after two bleaching sessions. With a significant level of 5%, statistic power of 80%, and considering an increase in the primary outcome measure from ‘‘11’’ in HP35 group to ‘‘9’’ in the BC group, a minimum of 14 samples each group would be required.

### Bleaching protocols

Both BC and WBC groups underwent toothbrushing in a brushing test machine (MAVTEC – Comércio e Serviços e Desenvolvimento para Laboratório, Ribeirão Preto, SP, Brazil). For each experimental set, the samples were fixed in the machine and brushed with a toothpaste corresponding to their group for 3 min *per* day to simulate the recommended toothbrushing technique for patients (3 times *per* day, 1 min each time). Each toothpaste was mixed in distilled water and in a sodium carboxymethyl cellulose solution at 0.5% (CMCS), in the toothpaste:water:CMCS ratio of 1:1:1, according to a method from Joiner, et al.^12^ ^,^ ^13^ (2010, 2008). The brushing test machine was programmed at 150 strokes/min, with a load of 375g, totaling 450 strokes *per* day. After toothbrushing, each set was washed in water to completely remove the toothpaste, and stored in artificial saliva.

The HP35 group was subjected to an in-office tooth bleaching protocol using 35% hydrogen peroxide (Whiteness HP, FGM Produtos Odontológicos, Joinville, SC, Brasil). The bleaching agent was prepared as recommended by the manufacturer. Two bleaching sessions were performed with a seven-day interval between them. In each of the bleaching sessions, the bleaching agent was applied for three sessions, 15 min each in duration. After each session, the sample sets were washed and placed in artificial saliva. The total time of contact between the bleaching agent and the dental enamel was 90 min.

The CP10 group was treated by an at-home tooth bleaching agent containing 10% carbamide peroxide. For this purpose, individual trays were fabricated with 1 mm acetate plaques (Whiteness, FGM Produtos Odontológicos, Joinville, SC, Brasil) to uniformly apply a 1 mm thick bleaching gel over each dental block. The tray and the respective set were placed in a container with artificial saliva and kept under gentle agitation at a temperature of 36±1^o^C for 4 h daily. Then, the bleaching agent was removed, and the sample was washed in water and stored in artificial saliva. This procedure was repeated for 14 consecutive days, and the total contact time of the bleaching gel with the dental structure was 3360 minutes. The control group samples remained in artificial saliva under stirring and controlled temperature (36±1.0°C) over the course of the experiment.

### Dental bleaching efficacy

The efficacy was measured by a reflectance spectrophotometer (Vita EasyShade^**®**^ – Vident, Brea, CA, USA) with a previously calibrated (ICC=0.74) and blinded examiner, using a reflectance spectrophotometer. The analyses were determined based on ∆L, ∆a, ∆b, and ∆E coordinates, from the CIELab system and assessed at T0 – baseline prior to treatment, T1 – immediately after toothbrushing or tooth bleaching treatments, T2 – 7 days after the conclusion of treatment, T3 - 14 days after the conclusion of treatment, and T4 – 21 days after the conclusion of the treatment. The dependent variable was the efficacy, and the independent variables were experimental groups and evaluation times. The latter was also the one that generated the repeated measures variable. To evaluate the effect of the treatments on the color difference, the difference between the color at T0 and subsequent records (T1, T2, T3 and T4) were considered. The following formula was used to calculate the bleaching efficacy, ∆E:∆E*=[(∆L*)^2^+(∆a*)^2^+(∆b*)^2^ ]^1/2^.

### Statistical analysis

Data for the color difference (∆E) and each CIELab color axis (L*, a*, and b*) were analyzed by a repeated measures mixed ANOVA. First, the conditions for the application of this test, namely normal distribution, sphericity, and homogeneity of variances, were tested through the Shapiro-Wilk, Mauchly, and Levene tests, respectively (p<0.05). The Mauchly test indicated the need for using the Greenhouse-Geisser correction (p<0.001). The Bonferroni test was used to identify which pairs of means differed for the interaction between the variables and experimental groups. All the statistical tests were performed with the assistance of the PAWS Statistics Software (PASW Statistics 21.0, SPSS Inc, Chicago, IL, USA), considering a type I error probability (α) of 0.05.

## RESULTS

Each experimental group color characteristics (L*, a*, and b*) at baseline - means ± standard deviation (SD) – are shown in Table 1.

**Table 1:** Means and standard deviation (± SD) of the baseline color characteristics for each experimental group

Experimental group	L*			a*			b*		
	mean	±	sd	mean	±	sd	mean	±	sd
C	77.7	±	5.7	1.4	±	6.8	19.4	±	4.6
BC	77.4	±	3.5	1.4	±	3.0	19.0	±	5.8
WBC	78.5	±	4.8	0.0	±	3.8	19.2	±	4.4
HP35	77.5	±	4.0	3.8	±	5.7	18.7	±	5.3
CP10	79.7	±	4.2	0.6	±	5.9	18.5	±	5.8

### Color difference (∆E)

The repeated measures mixed ANOVA showed significance for experimental groups, and the interaction between evaluation times and experimental groups (p<0.001). Since there was significance between the interaction of the factors, a *post hoc* Bonferroni test was performed and demonstrated significant differences between the groups CP10 and HP35 for the treatments C, BC, and WBC. The means and confidence intervals (95% CI) are shown in Table 2.

**Table 2:** Mean and confidence intervals (95% CI) of color difference (ΔE) for each experimental group at all evaluation time points. *Different letters indicate significant differences (p<0.05)

Evaluation time	Experimental group									
	C		BC		WBC		HP35		CP10	
	mean	CI 95%	mean	CI 95%	mean	CI 95%	mean	CI 95%	mean	CI 95%
T1	5.9^b^	1.8	4.2^b^	1.8	5.4^b^	1.8	8.3^ab^	1.8	11.0^a^	1.8
T2	4.9^b^	1.8	4.0^b^	1.8	5.2^b^	1.8	11.7^a^	1.8	10.4^a^	1.8
T3	4.3^b^	1.8	4.2^b^	1.8	6.8^ab^	1.8	10.7^a^	1.8	10.5^a^	1.8
T4	8.7^ab^	1.8	4.2^b^	1.8	4.1^b^	1.8	10.4^a^	1.8	10.2^a^	1.8

*Different letters indicate significant differences (p<0.05)

### Luminosity (∆L)

Significance was found for the factors experimental group (p<0.001) and evaluation time (p=0.006). The interaction between factors showed up at the significance threshold (p=0.054). The Bonferroni test demonstrated statistically significant differences between groups WBC, HP35 and groups CP10, C and BC. Table 3 shows means and confidence intervals (95% CI).

**Table 3:** Means of luminosity (ΔL), green-red axis (Δa), and blue-yellow axis (Δb) for each experimental group at all evaluation time points. *Different letters indicate significant differences (p<0.05). Confidence intervals of luminosity (ΔL), green-red axis (Δa), and blue-yellow axis (Δb) were 1.8, 1.3 and 1.6, respectively

Evaluation time	Experimental group														
	C			BC			WBC			HP35			CP10		
	ΔL	Δa	Δb	ΔL	Δa	Δb	ΔL	Δa	Δb	ΔL	Δa	Δb	ΔL	Δa	Δb
T1	-1.8^fg^	-1.6^a^	-3.8^de^	0.8^defg^	-1.2^a^	-1.9^e^	3.4^abcd^	-1.4^a^	-2.2^e^	4.5^abc^	-0.2^a^	-6.2^cd^	6.3^ab^	-1.3^a^	-8.1^abc^
T2	-1.8^fg^	-2.9^a^	-2.3^e^	0.6^efg^	-1.9^a^	-1.7^e^	2.8^bcde^	-1.9^a^	-3.0^de^	2.0^cde^	-1.4^a^	-10.5^a^	5.1^abc^	-1.7^a^	-8.0^abc^
T3	-0.2^efg^	-2.7^a^	-1.3^e^	1.7^def^	-1.6^a^	-1.2^e^	3.6^abcd^	-1.2^a^	-1.4^e^	3.2^abcde^	-1.7^a^	-9.7^ab^	6.6^a^	-1.4^a^	-7.1^bc^
T4	-2.4^g^	-2.7^a^	-0.4^e^	-0.5^fg^	0.0^a^	1.3^f^	2.4^cde^	-1.1^a^	-1.4^e^	5.0^abc^	-1.3^a^	-8.4^abc^	4.3^abc^	-1.9^a^	-8.0^abc^

*Different letters indicate significant differences (p<0.05). Confidence intervals of luminosity (ΔL), green-red axis (Δa) and blue-yellow axis (Δb) were 1.8, 1.3 and 1.6, respectively

### Green-red axis (∆a)

Despite the results from the repeated measures mixed ANOVA, which demonstrate statistically significant differences in the significance threshold for time evaluation (p=0.049), the Bonferroni post-test found no significant difference between the evaluation times. The means and confidence intervals (95% CI) are shown in Table 3.

### Blue-yellow axis (∆b)

The repeated measures mixed ANOVA showed significant differences for the factors evaluation time, experimental group, and the interaction between factors (p<0.001). Overall, the Bonferroni test demonstrated that treatments HP35 and CP10 were significantly different than treatments C, BC, and WBC. Table 3 shows means and confidence intervals (95% CI).

## DISCUSSION

Several studies in tooth bleaching using peroxide based products have shown that the yellow-blue axis is the most important factor for the perception of tooth bleaching^6^ ^,^ ^7^. Furthermore, a reduction in the b* value occurs more quickly and to a higher extent than reductions in the L* value^9^ ^,^ ^15^. This observation was applied in developing the bleaching toothpaste containing Blue Covarine^11^ ^,^ ^13^. The “optical” bleaching effect of this toothpaste is based on the change in the perception of the original color of the teeth, due to deposition of a thin blueish color film on the tooth structure after brushing. The presence of the film should reduce the axis b* initial value (blue-yellow), lessening the yellow appearance of teeth. This toothpaste also has a dentifrice abrasive system for the removal of extrinsic stains compared to other whitening toothpastes based on silica^14^. However, the results of this study showed no evidence of a bleaching effect when using the toothpaste containing Blue Covarine. The analysis of the parameters ΔE, ΔL, Δa, and Δb were not identified as statistically significant differences between the toothpaste containing Blue Covarine group, the control group or a toothpaste without Blue Covarine.

Torres, et al.^18^ (2013) also found no difference in the parameters of ΔE, ΔL, and Δb. A similarity between our current study and the study of Torres, et al.^18^ (2013) was the use of a spectrophotometer for color measurement, whereas studies that prove the presence of the thin film Blue Covarine evaluated the color alteration by digital images^2^ or a colorimeter^1^ ^,^ ^13^ ^,^ ^14^. These methods measure the color from the tooth surface, and therefore may have a greater sensitivity to show the presence of a Blue Covarine film. The reflectance spectrophotometer Vita Easyshade Advance, used in this study, is designed specifically for color analysis of teeth, ceramics and other translucent materials. This instrument is programmed to record the color of dentin and deep layers of enamel, ignoring the reflection and the surface irregularities of the enamel. Therefore, this may be responsible for the lack of an apparent bleaching effect on the specimens analyzed, as the Blue Covarine film, responsible for the optical bleaching effect, is deposited only on the surface of teeth, and may not be effectively detected by spectrophotometric methods. Another reason is that the acquired pellicle may not have been properly formed, because the specimens used in this study were stored in artificial saliva, whereas in other studies human saliva was used, having an indication of a strong affinity of the Blue Covarine with some components of the acquired pellicle^1^ ^,^ ^13^.

Except for Δa, groups C, BC, and WBC were significantly different from groups HP35 and CP10 for all other parameters analyzed. The latter groups were used as positive controls because they are substances with proven efficacy in tooth bleaching. Thus, the null hypothesis was rejected because the bleaching toothpaste containing Blue Covarine did not promote tooth bleaching similar to at-home and in-office techniques. As previously demonstrated, in-office and at-home bleaching products promoted similar bleaching, which remained in the T4 analysis, demonstrating that both techniques are effective and stable for at least 21 days after the end of the treatment.

## CONCLUSION

Within the limitations of this *in vitro* study, it can be concluded that the use of bleaching toothpastes either with or without Blue Covarine is significantly less effective at whitening teeth than either an in-office 35% hydrogen peroxide or an at-home 10% carbamide peroxide bleaching agent. Furthermore, the bleaching toothpaste with Blue Covarine did not show a statistically significant improvement over a conventional bleaching toothpaste without Blue Covarine, and neither of these bleaching toothpaste were more effective than the control.
